# Loss of function Cbl-c mutations in solid tumors

**DOI:** 10.1371/journal.pone.0219143

**Published:** 2019-07-01

**Authors:** Silvano Rakeem Daniels, Mariya Liyasova, Stephen C. Kales, Marion M. Nau, Philip E. Ryan, Jeffrey E. Green, Stanley Lipkowitz

**Affiliations:** 1 Women’s Malignancies Branch, Center for Cancer Research, National Cancer Institute, Bethesda, Maryland, United States of America; 2 Laboratory of Cancer Biology and Genetics, Center for Cancer Research, National Cancer Institute, Bethesda, Maryland, United States of America; Hungarian Academy of Sciences, HUNGARY

## Abstract

Receptor Tyrosine Kinase (RTK) signaling is essential for normal biological processes and disruption of this regulation can lead to tumor initiation and progression. Cbl proteins (Cbl, Cbl-b and Cbl-c) are a family of RING finger (RF) ubiquitin ligases that negatively regulate a variety of RTKs, including EGFR, MET, and RET. Recent studies have identified Cbl mutations associated with human myeloid neoplasias in approximately 5% of the cases. Cbl-c is the most recently identified human Cbl protein and is expressed exclusively in epithelial cells. We identified a novel cDNA that was isolated from a mouse mammary cancer from the C3(1) Large T Antigen transgenic model. This mutant cDNA encodes a protein that has a deletion in the RF domain of Cbl-c, thereby resembling known Cbl family mutations associated with myeoloid neoplasias. Genomic analysis of both parental and transgenic lines shows no evidence of germline mutation indicating that this mutation is likely a somatic mutation. The mutant protein enhances transformation of NIH 3T3 cells when expressed in combination with SV40 Large T antigen. Together these data are consistent with a second hit mutation. In overexpression studies, this mutant Cbl-c protein fails to mediate ubiquitination of activated EGFR and acts in a dominant negative fashion to prevent ubiquitination and downregulation of the activated EGFR by wild type Cbl proteins. Mechanistically, the mutant Cbl-c binds to the EGFR and prevents recruitment of the wild type Cbl protein. Furthermore, data mining reveals Cbl-c mutations associated with solid tumors in humans. Subsequent cell-based analysis demonstrates a similar loss of E3 function and dominant negative effects for one of these human mutations. These data suggest that like Cbl mutations in myeloid neoplasms, loss of Cbl-c function may contribute to the pathogenesis of solid tumors in murine models and in humans.

## Introduction

Cbl proteins are a family of RING Finger (RF) ubiquitin ligases that regulate signaling by many tyrosine kinases (reviewed in [1]). Cbl proteins are recruited to many activated receptor tyrosine kinases (RTKs) where they mediated mono-ubiquitination and K63-linked di-ubiquitination of the RTK, leading to trafficking of the ubiquitinated receptor/Cbl complex to the lysosome for degradation [1–5]. v-Cbl was originally identified as an oncogene, causing leukemia in mice and transforming NIH 3T3 cells [6, 7]. The v-Cbl protein contains only the tyrosine kinase binding (TKB) domain of Cbl and, when expressed in cells, prevents RTK ubiquitination and downregulation most likely by acting as a dominant negative protein, preventing the recruitment of endogenous Cbl proteins to the RTK [8, 9]. Two other transforming forms of the murine Cbl protein have been identified from a chemically induced murine pre-B cell lymphoma and a chemically induced histiocytic lymphoma (70Z Cbl and p95 Cbl, respectively) [10, 11]. These mutants both contain deletions within the linker and RF regions, and thus these proteins have lost ubiquitin ligase (E3) activity [10, 11]. More recently, Cbl mutations that disrupt E3 function have been found in ~5% of a wide variety of myeloid neoplasms including myelodysplastic syndrome, myelofibrosis, refractory anemia with excess blasts, de novo and secondary acute myeloid leukemia (AML and sAML, respectively), atypical chronic myelogenous leukemia (aCML), CML in blast crisis, chronic myelomonocytic leukemia (CMML), and juvenile myelomonocytic leukemia (JMML) (reviewed in [12]). The majority of these mutations are missense mutations that cluster within the linker region and at or near the zinc coordinating amino acids within the RF domain. In addition, deletions of all or portions of the Cbl exon containing the distal portion of the linker region and the proximal portion of the RF have been described [12]. Similar to v-Cbl, these mutated proteins act in a dominant negative fashion by blocking recruitment of the WT Cbl protiens to the activated receptors [12], Thus, mutations of Cbl contribute to the development of human myeloid neoplasms.

Cbl-c is the most recently identified Cbl protein and is expressed exclusively in epithelial cells while Cbl and Cbl-b are widely expressed in mammalian tissues [13–16]. Like Cbl and Cbl-b, the N-terminus of Cbl-c is composed of the highly-conserved TKB and RF separated by an alpha helical linker region [14, 17]. The C-terminus of the Cbl-c protein is shorter and diverges significantly from Cbl and Cbl-b [14]. Cbl-c, like Cbl and Cbl-b, is a functional E3 that can ubiquitinate and downregulate the EGFR and other tyrosine kinases such as v-Src, and RET [14, 18–21]. The expression in epithelial cells and function as an E3 for tyrosine kinases suggests that mutations of Cbl-c could contribute to the development of epithelial malignances.

Here we describe a mutant form of Cbl-c found in a murine mammary cancer model driven by SV40 Large T antigen and demonstrate that the mutant protein lacks E3 activity, acts in a dominant negative fashion to prevent ubiquitination of activated EGFR by wild type Cbl proteins, and can enhance transformation by Large T antigen. Further, mutations in Cbl-c are catalogued in the somatic mutation databases from human tumors and several of these also abrogate Cbl-c E3 activity and convert Cbl-c into a dominant negative protein. Thus, like mutations of Cbl in myeloid neoplasms, mutations of Cbl-c may contribute to development of solid tumors.

## Materials and methods

### Reagents

Dulbecco’s Modified Eagle’s Medium (DMEM), Fetal Bovine Serum (FBS), penicillin, and streptomycin sulfate were obtained from Invitrogen (Carlsbad, CA). Dulbecco’s Phosphate Buffered Saline (D-PBS) was purchased from Mediatech Inc. (Herndon, VA). Tissue culture plastic ware and other laboratory consumables were purchased from commercial sources. Recombinant human EGF (#354052) was obtained from BD Biosciences (Bedford, MA).

### Expression constructs

Plasmids encoding wild-type and mutant mouse Cbl-c were obtained from Open Biosystems (GE Dharmacon (RNA accessions BC111037 and BC046337, respectively). Expression plasmids for full length human GST-tagged Cbl-c, GFP-tagged Cbl-c, HA-tagged c-Cbl, and the control vector (pCEFL) have been described previously [14, 22]. Plasmid encoding SV40 large T antigen was obtained from F.S. Kaye and sub-cloned into pcDNA 3.1. Point mutation constructs described here were created using the QuikChange II Site-directed Mutagenesis Kit according to manufacturer’s instructions (Stratagene, La Jolla, CA). All constructs were confirmed by DNA sequencing prior to use.

### Cell culture and transfections

All cell lines used in this study were originally obtained from ATCC and maintained in our laboratory through regular passage and cryopreservation. The human embryonic kidney (HEK 293T) and mouse fibroblast (NIH 3T3) cell lines were maintained in culture using DMEM (Invitrogen, Carlsbad, CA) supplemented with 10% FBS, 100 U/ml penicillin, and 100 μg/ml streptomycin sulfate. HEK 293T cells were transfected using calcium phosphate (Profection; Promega Corp., Madison, WI). Transfections were incubated 18 h prior to media change and grown for a total of 48 h prior to harvesting. For cell treatments, all transfections were performed in replicate, then pooled, and re-plated prior to treatment. To test EGFR ubiquitination, cells were starved for 3 h in DMEM media without FBS and then either left untreated (t = 0) or treated with 100 ng/ml EGF (BD Biosciences, Bedford, MA) for 10 or 30 min prior to harvesting. Each cell-based experiment was repeated at least 3 times. NIH 3T3 cells were transfected using Lipofectamine 2000 according to manufacturer’s instructions (Life Technologies, Grand Island, NY).

### Sequence and Southern analysis

Genomic DNA (gDNA) from the parental FVB/N and transgenic C3(1) SV40 Large T antigen (Tag) mouse strain and RNA from 21 murine tumors that developed in the transgenic mice were prepared using TRIzol reagent (ThermoFisher Scientific, Waltham, MA) as recommended by the supplier. For Southern analysis, the gDNA was digested with the NCO1 restriction enzyme (Life Technologies, Grand Island, NY), run on 0.8% agarose gels, and transferred to nitrocellulose filters [23]. The filters were hybridized with digoxigenin labeled cDNA using the DIG Easy Hybe Kit (Roche Applied Sciences, Penzberg, Germany), and signal was detected using CDP-Star ready-to-use detection kit (Roche Applied Sciences, Penzberg, Germany). RT-PCR for murine Cbl-c was performed on the isolated RNA as previously described [14] and the entire cDNA was sequenced. The plasmids encoding the wild type and mutant murine Cbl-c cDNAs and the products of the RT-PCR were sequenced by Sanger sequencing in the Center for Cancer Research Genomics Core.

### Immunoblotting and immunoprecipitation

To harvest proteins, cells were washed twice in ice-cold Dulbecco’s-PBS and then lysed in ice-cold lysis buffer (10 mM Tris-HCl pH 7.5, 150 mM NaCl, 5 mM EDTA, 1% Triton X-100, 10% glycerol, 2 mM sodium orthovanadate, and protease inhibitors [Complete tabs, Roche Diagnostics Corp., Indianapolis, IN]). All whole cell lysates were cleared of cellular debris by centrifugation at 16,000 × *g* for 15 m at 4°C. Supernatant protein concentrations were determined using the Bio Rad protein assay (BioRad, Hercules, CA). For immunoblotting, lysates (2 μg protein/μl) were boiled in 2X loading buffer (62.5 mM Tris-HCl pH 6.8, 10% glycerol, 2% SDS, 1 mg/ml bromphenol blue, 0.3573 M β-mercaptoethanol) for 5 min then resolved by SDS-PAGE and transferred to nitrocellulose membranes (Protran BA85; Whatman, Sanford, MA). Separate lanes included protein molecular weight standards according to manufacturer’s instructions (BioRad, Hercules, CA). For immunoprecipitations, 300–1500 μg of each of the whole cell lysates were incubated with rabbit anti-EGFR (Ab-3; Millipore Sigma, Burlington, MA) and Protein A/G^+^ agarose beads (sc-2003; Santa Cruz Biotechnology, Dallas, Tx). All immunoprecipitations were incubated overnight at 4°C with tumbling. Immune complexes were washed five times in 1mL cold lysis buffer, and then boiled in 2X loading buffer for 5 min prior to electrophoresis and blotting as described above.

### Antibodies

The following antibodies were used to detect proteins of interest by immunoblotting: rabbit anti-EGFR (#2232, Cell Signaling Technololgy, Danvers, MA), mouse anti-GST (sc-138; Santa Cruz Biotechnology, Dallas, TX), mouse anti-Hsc70 (sc-7298, Santa Cruz Biotechnology, Dallas, TX); rabbit anti-Cbl (sc-170; Santa Cruz Biotechnology, Dallas, TX), mouse anti-ubiquitin (sc-8017; Santa Cruz Biotechnology, Dallas, TX), rabbit anti-GFP (sc-8334; Santa Cruz Biotechnology, Dallas, TX), rat monoclonal high affinity anti-HA-peroxidase, (clone 3F10; Roche Life Science, Penzburg, Germany), and rabbit anti-Cbl-c (13104-1-AP; ProteinTech Group, Chicago, IL). Mouse anti-EGFR (Ab-3; Millipore Sigma, Burlington, MA) and Protein A/G+ agarose beads (sc-2003; Santa Cruz Biotechnology, Dallas, TX) were used for immunoprecipitation of EGFR. Glutathione-agarose beads (sc-2009; Santa Cruz Biotechnology, Dallas, TX) were used for the pull-down of GST-tagged mutant Cbl-c. Horseradish peroxidase linked donkey anti-rabbit IgG (NA934V; GE Healthcare, Piscataway, NJ) or horseradish peroxidase linked donkey anti-mouse IgG (NA931: GE Healthcare, Piscataway, NJ) was used with SuperSignal reagents (Pierce Biotechnology Inc., Rockford, IL) to visualize the blots. The Li-Cor Odyssey blot imager (Li-Cor Inc., Lincoln, NE) and Image Studio software were used to detect and quantify Western blot data. Each experiment was repeated at least 3 times.

### Cell transformation assays

Transformation assays were performed according to a modified protocol described previously [24]. Briefly, NIH 3T3 cells were plated at 1.0x10^6^/100mm dish and allowed to adhere overnight. Attached cells were then transfected, as indicated, using 6 μgs of plasmid encoding either wild-type or mutant mouse Cbl-c with and without 1 μg of plasmid encoding SV40 T antigen. All transfections were balanced with empty vector controls as indicated. At 24 h post-transfection, cells were split 1:3 and maintained in culture for 3 weeks with bi-weekly media changes then fixed and stained with 2% Methylene Blue in 50% Methanol prior to scoring for foci formation using an AlphaInnotech imager (R&D Systems, Minneapolis, MN).

### GST pull-downs

HEK 293T cells were transfected with combinations of GST, HA-tagged WT Cbl-c, and GST-tagged mutant Cbl-c by using calcium phosphate, as described above. Cells were incubated at 37°C for 16 hours and given time to recover in DMEM media (10% FBS) for 24 hours prior to harvesting protein lysates. Cells were lysed and protein were quantified as described above. Cell lysate (700 μg protein) was combined with 40 μL glutathione-agarose (sc-2009; Santa Cruz Biotechnology, Dallas, TX) in microcentrifuge tubes. Cold lysis buffer was added to a total volume of 500 μL and samples were incubated overnight at 4°C with tumbling. Complexes (Glutathione-agarose + GST-tagged mutant Cbl-c) were washed a total of five times in cold lysis buffer, resuspended in 2X loading buffer, and boiled for 7 minutes prior to gel electrophoresis and blotting, as described above.

### Statistics

Student’s t-tests were used to analyze statistical significance, measured by p-values, of EGFR downregulation, ubiquitination, and recruitment of Cbl to EGFR. Plotted error bars indicate standard error of the mean (SE).

### Database searches

Mutations in the three Cbl genes were identified using the cBioPortal for Cancer Genomics [25, 26]. Searches of human cancer data were performed and were restricted to mutations. Searches eliminated redundant data and did not include cell lines, patient derived xenografts, or other samples that were not direct analysis of patient samples.

## Results

**Fig 1:**
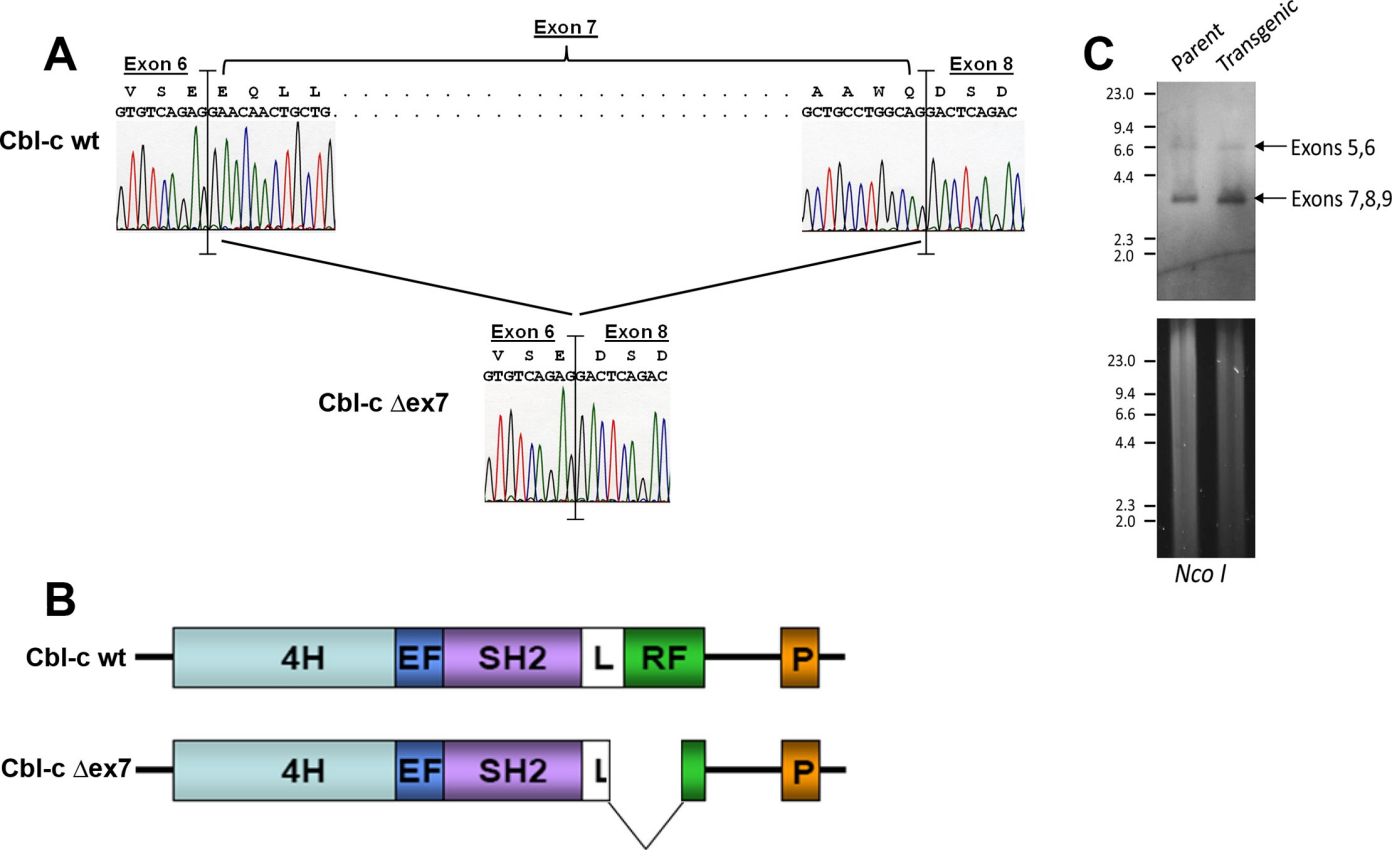
Structure of a Cbl-c mutant (Cbl-c Δex7) isolated from a mouse ductal carcinoma. (A) Sequence analysis of Cbl-c wildtype and Cbl-c Δex7. Sequence for wild type cDNA is shown on top of panel and sequence for mutant is shown on bottom demonstrating an in-frame deletion of exon 7 which encodes part of the linker domain and the catalytic RING finger domain. (B) Cartoon depicting structural comparison of mouse wild-type and mutant mouse Cbl-c Δex7. (C) Genomic DNA from parental and transgenic mouse stocks were digested, as indicated, separated by electrophoresis, stained with cyber green (bottom panel), then transferred to nylon membrane (upper panel) and probed to detect regions flanking exon 7 of mouse Cbl-c. Arrows indicate the bands containing exons 5 and 6 and the band containing exons 7-9.DNA molecular weight standards in kilobases are indicated on the left of each panel.

While searching online databases for a murine Cbl-c cDNA expression construct, we identified a mutant form of the murine Cbl-c cDNA in the Dharmacon Mammalian Gene Collection Clones Database which was predicted to contain an in-frame deletion of the linker and first part of the Ring Finger (RF) (Cbl-c Δex7) [27]. We obtained the cDNA from the Dharmacon Mammalian Gene Collection Clones Database and confirmed that the construct does contain this in-frame deletion (Fig 1A and 1B). This mutant Cbl-c was isolated from a mammary carcinoma that developed in the C3(1) SV40 Large T antigen (Tag) transgenic mouse model of breast cancer [28, 29]. Unfortunately, the mouse tumor from which this cDNA was isolated was no longer available for further analysis of the germline and tumor DNA to determine the nature of this mutation. To examine whether the mutation was likely a somatic mutation, we performed Southern analysis of genomic DNA (gDNA) from the parental and C3(1) Tag transgenic strains of the FVB/N mice used for this model. Southern analysis of the gDNA revealed that the parental and transgenic mice had identical hybridization patterns for exons 5–9 which is inconsistent with a germline deletion in exon 7 (Fig 1C). Similar in-frame deletions of the linker region and RF have been reported in Cbl in murine lymphomas and human myeloid neoplasms [12]. These mutations often arise from missplicing due to mutations, small insertions, or small deletions within the splice acceptor site for the Cbl exon 8 (equivalent to Cbl-c exon 7) [12, 30]. We therefore sequenced exon 7 and the surrounding intronic DNA from the gDNA from the parental and transgenic stains of FVB/N mice but did not find any evidence for mutations, small insertions, or small deletions in the gDNA. Although not definitive without the ability to analyze the tumor and germline DNA from the affected mouse, these data are most consistent with the Cbl-c deletion mutation arising as a somatic mutation in the tumor in which it was found. We examined the Cbl-c cDNAs from 21 additional mammary tumors from the C3(1) Tag mice but no other mutations in Cbl-c were identified suggesting that this is a low frequency event.

**Fig 2:**
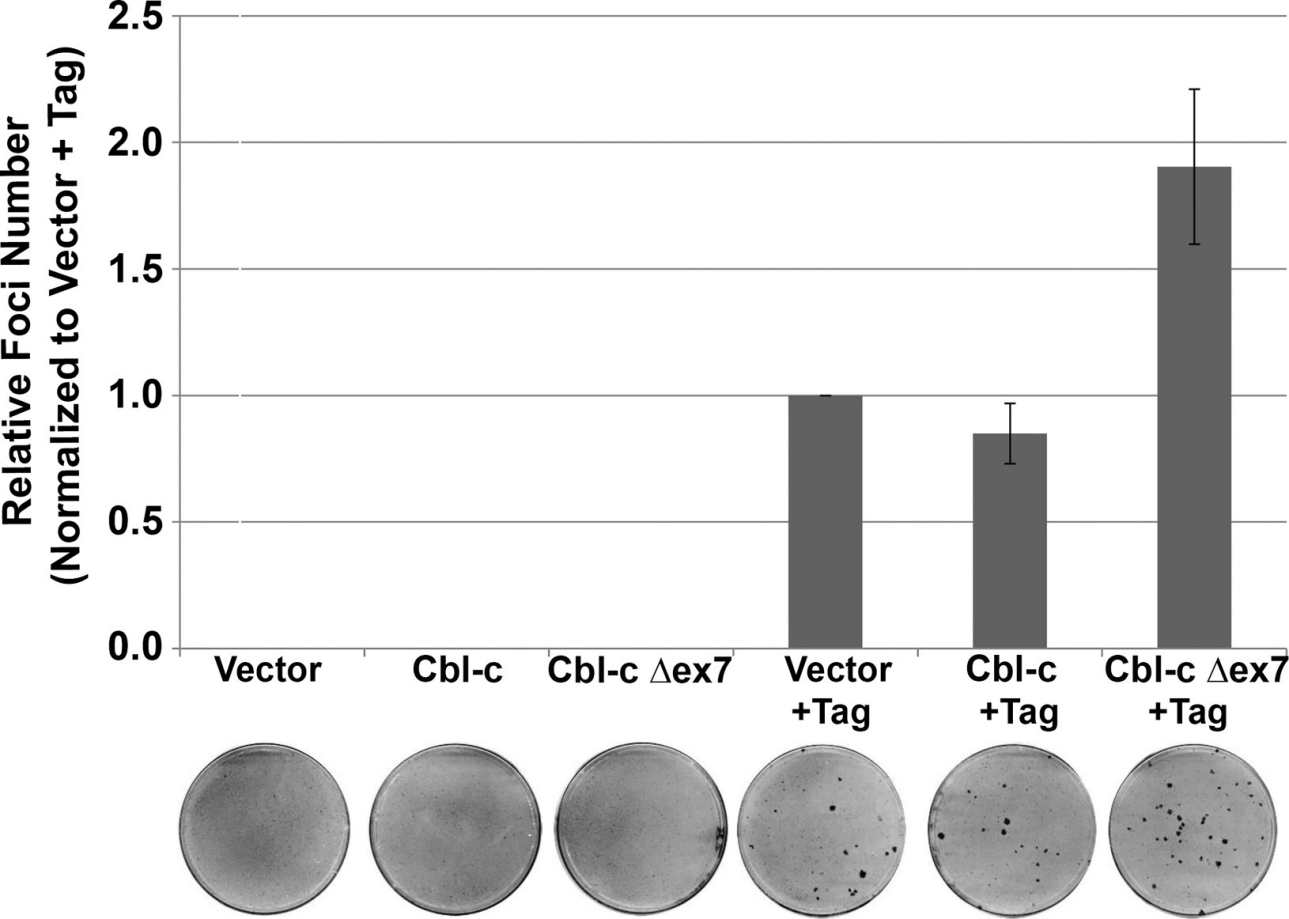
Mutant Cbl-c Δex7 enhances cell transformation by SV40 T antigen. NIH 3T3 cells were transfected with plasmids encoding either wild-type or mutant Cbl-c both with and without SV40 Large T Antigen. Cells were maintained with bi-weekly media changes for 21 days prior to staining. Foci per plate relative to Tag alone are shown for 3 independent experiments as the mean +/- SE. Representative plates of each transfection are shown below the graph.

As the Cbl-c mutant was found in a tumor initiated by expression of SV40 Tag, we tested the ability of the mutant to transform NIH 3T3 cells alone or in combination with Tag. Neither Cbl-c wild-type (WT) nor Δex7 led to foci formation by the NIH3T3 cells (Fig 2; plates 2 and 3 respectively). Expression of Tag resulted in foci formation (Fig 2; plate 4) and when Cbl-c Δex7 was co-expressed along with Tag, it increased the number of transformed colonies by approximately two-fold (Fig 2; plate 6). Co-expression of WT Cbl-c along with Tag did not significantly change the number of foci (Fig 2, plate 5). Thus, these data are consistent with the Cbl-c mutant acting as a second hit to promote transformation in the murine mammary tumors.

**Fig 3:**
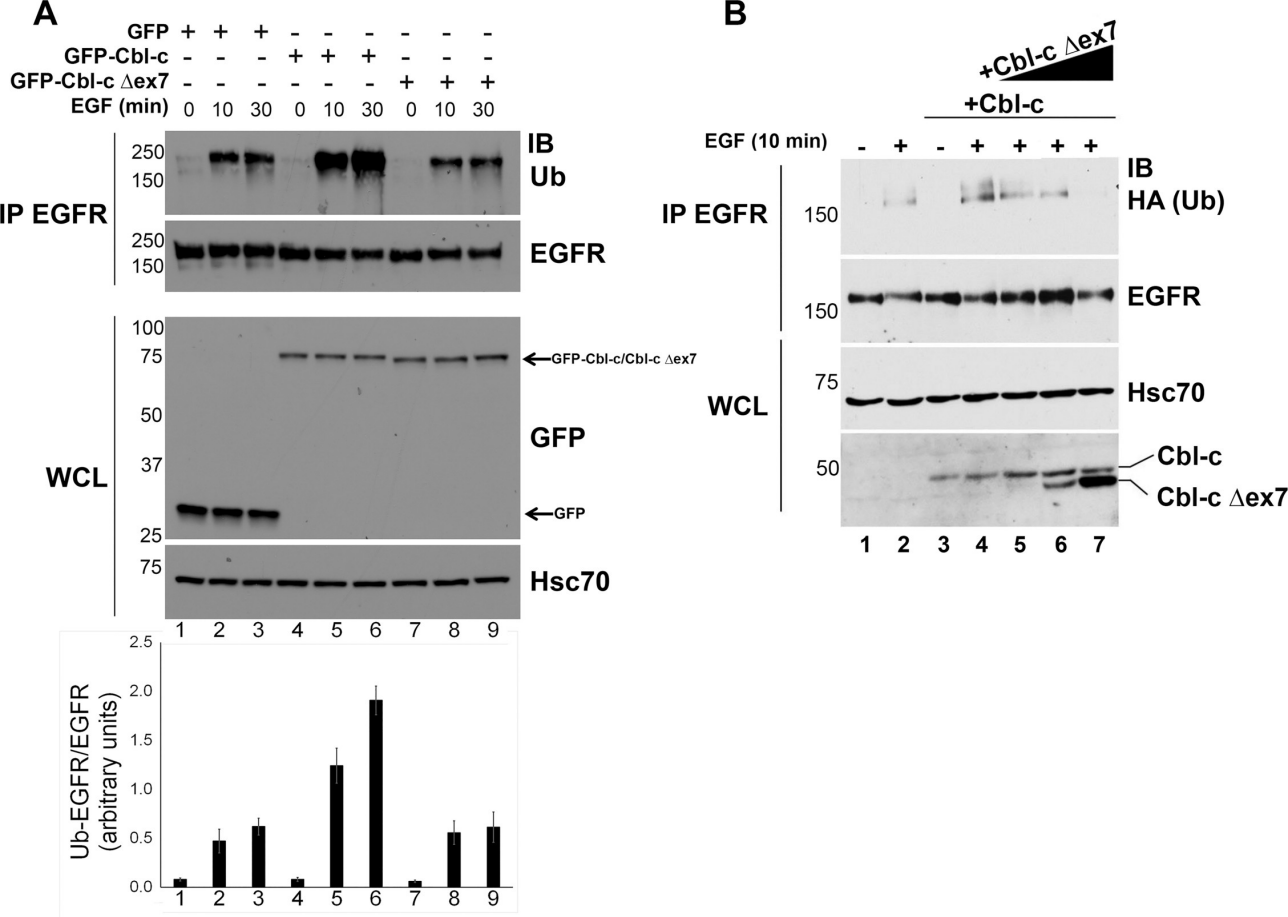
Loss of E3 function in a mouse ductal carcinoma Cbl-c mutant. **(**A**)** Cbl-c Δex7 lacks E3 activity. HEK 293T cells were transfected with plasmid encoding wild-type and mutant mouse GFP-tagged Cbl-c along with human EGFR. Triplicate plates were pooled and split at 24 h post-transfection. At 48 h post-transfection, triplicate plates were treated with 100 ng/mL EGF for 10 or 30 min prior to cell harvesting. EGFR immunoprecipitations were immunoblotted for endogenous Ub to visualize EGFR ubiquitination. The immunoblots were quantified by image analysis and the intensity of the ubiquitinated EGFR/total EGFR is shown in the graph below the immunoblot. The graph represents the average relative ubiquitination/EGFR+/- SE for 5 experiments. (B) Cbl-c Δex7 acts to prevent ubiquitination of the activated EGFR by wild type Cbl-c. HEK 293T cells were transfected with plasmid encoding wild-type and mutant mouse Cbl-c along with human EGFR and HA-tagged ubiquitin as indicated. Duplicate plates were pooled and split at 24 h post-transfection. At 48 h post-transfection, duplicate plates were treated with EGF (or water as a vehicle control) for 10 min prior to cell harvesting. EGFR immunoprecipitations were immunoblotted for HA to visualize EGFR ubiquitination. Whole cell lysates were immunoblotted and probed for mouse Cbl-c with either anti-GFP or anti-Cbl-c antibodies and human Hsc70 which served as a lysate loading control. Molecular weight standards in kDa are indicated on the left of each panel.

The deletion of the linker and RF of Cbl-c would be predicted to abrogate E3 activity, as seen in such mutants of Cbl [12]. Cbl proteins have been shown to ubiquitinate and negatively regulate many RTKs and the EGFR is a well characterized substrate of Cbl protein function [1]. We tested the ability of WT and Δex7 Cbl-c to ubiquitinate the EGFR. GFP tagged constructs of murine wildtype and Δex7 Cbl-c were transfected into HEK 293T cells along with the EGFR, the cells were starved overnight, and then incubated with or without EGF (100 ng/ml). WT GFP-Cbl-c induced 2–3 fold increased ubiquitination 2–3 fold upon EGF stimulation compared to the GFP control transfected cells (Fig 3A; compare lanes 4–6 to lanes 1–3 and quantification below the immunoblot). By contrast, GFP-Cbl-c Δex7 did not increase ubiquitination of the EGFR over the GFP control transfected cells (Fig 3; compare lanes 7–9 to lanes 1–3 and quantification below the immunoblot). To test whether the Cbl-c Δex7 could act in a dominant negative manner against WT Cbl-c, 293T cells were transfected with WT Cbl-c, along with the EGFR, in the absence or presence of increasing concentrations of Cbl-c Δex7. The cells were starved overnight, and then incubated with or without EGF (100 ng/ml). WT Cbl-c led to increased ubiquitination of the activated EGFR compared to empty vector (Fig 3B; compare lane 2 to lane 4). With increasing concentrations of cotransfected Cbl-c Δex7, there was decreased ubiquitination of the EGFR upon EGF stimulation. This is consistent with the mutant Cbl-c acting in a dominant negative manner to prevent ubiquitination of the EGFR by ectopic WT Cbl-c protein.

**Fig 4:**
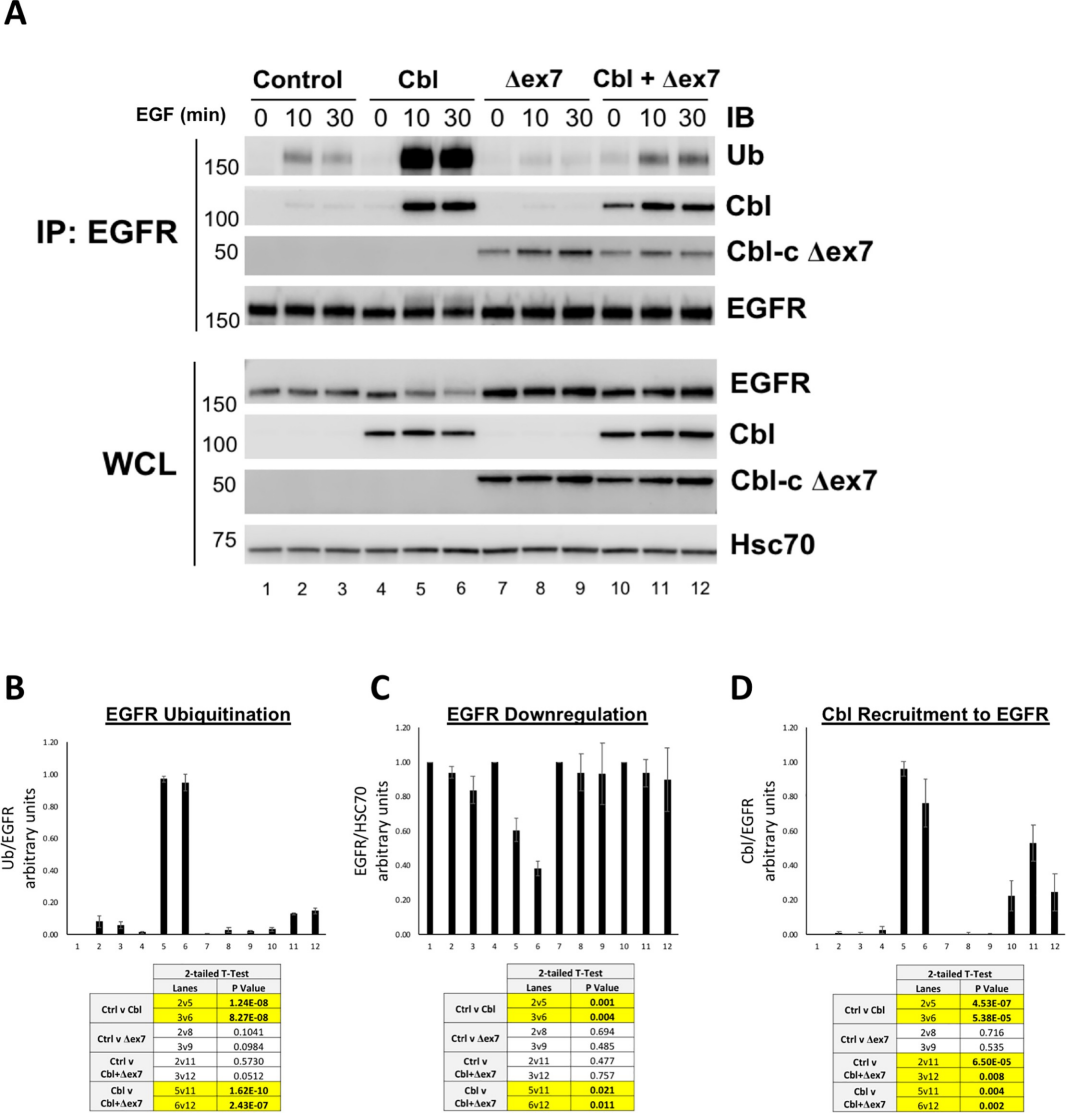
Murine mutant Cbl-c Δex7 prevents ubiquitination and downregulation of EGFR by WT Cbl proteins. (**A**) Cbl-c Δex7 prevents ubiquitination of EGFR by WT Cbl. HEK 293T cells were transfected with empty vector (Control), WT Cbl, Cbl-c Δex7, or a combination of WT Cbl and Cbl-c Δex7 as indicated. All cells were cotransfected with EGFR. Cells were stimulated with EGF for either 0, 10 or 30 minutes, harvested, and immunoprecipitated with anti-EGFR antibodies. The EGFR IP and WCL were probed for proteins as indicated. Molecular weight standards in kDa are indicated on the left of each panel. (**B**) EGFR-associated ubiquitin was quantified and normalized to total EGFR from the EGFR IP. (**C**) EGFR levels were quantified from the WCL and normalized to Hsc70. (**D**) EGFR-associated Cbl was quantified and normalized to total EGFR from the EGFR IP (Panel 2). Graphs represent the mean from 5 experiments +/- SE. p-values for relevant comparisons are shown in the tables accompanying the graphs. Significant comparisons (p≤0.05) are highlighted in yellow.

To test if Cbl-c Δex7 can inhibit ubiquitination of EGF-stimulated EGFR by other Cbl proteins, HEK 293T cells were cotransfected with control vector, WT Cbl, Cbl-c Δex7, or a combination of WT Cbl and Cbl-c Δex7 (Fig 4). Cells were stimulated with EGF (100 ng/mL), for either 10 or 30 minutes, or left unstimulated (0 min) and analyzed by immunoblot as described above. Ubiquitination of the EGFR and Cbl associated with immunoprecipitated EGFR was quantified, normalized to total EGFR (IP: EGFR), and averaged from three experimental repeats (Fig 4). Upon EGF treatment, WT Cbl induced a statistically significant increase in ubiquitination of the transfected EGFR compared to the vector controls (Fig 4A compare lanes 5 and 6 to lanes 2 and 3, quantified in Fig 4B). Again, as seen in Fig 3A, Cbl-c Δex7 did not significantly increase ubiquitination of the EGFR compared to the control lanes (Fig 4A, compare lanes 8 and 9 to lanes 2 and 3, quantified in Fig 4B). When WT Cbl and Cbl-c Δex7 were cotransfected, there was significantly less ubiquitination of the EGFR compared to the WT Cbl alone (Fig 4A, compare lanes 11 and 12 to lanes 5 and 6, quantified in Fig 4B).

In cells transfected with WT Cbl, there was an increase in the downregulation of the EGFR upon stimulation compared to the control cells (Fig 4A, fifth panel, compare lanes 4–6 to lanes 1–3, quantified in Fig 4C). Cbl-c Δex7 did not significantly change EGFR downregulation compared to control cells (Fig 4A, fifth panel, compare lanes 7–9 to lanes 1–3, quantified in Fig 4C). Importantly, the increased downregulation of the EGFR induced by expression of WT Cbl alone was blocked in the presence of Cbl-c Δex7 and was not significantly different from the control cells (Fig 4A, fifth panel, compare lanes 10–12 to lanes 4–6 and 1–3 respectively, quantified in Fig 4C).

In cells transfected with WT Cbl and EGFR, Cbl was recruited to the EGFR upon EGF stimulation (Fig 4A, lanes 4 and 5 in the second panel, quantified in Fig 4D). Cbl-c Δex7 was also recruited to the activated EGFR (Fig 4A, see lanes 7–12). When Cbl-c Δex7 Cbl-c was cotransfected with WT Cbl, there was a significant decrease in the amount of WT Cbl recruited to the activated EGFR (Fig 4A, compare Cbl recruitment in lanes 5 and 6 to lanes 11 and 12 in the second panel, quantified in Fig 4D).

Together, these results indicate that Cbl-c Δex7 significantly inhibits ubiquitination and downregulation of the activated EGFR by WT Cbl. Mechanistically, we conclude that this is due to inhibition of WT Cbl recruitment to the activated EGFR by the mutant Cbl-c Δex7.

**Fig 5:**
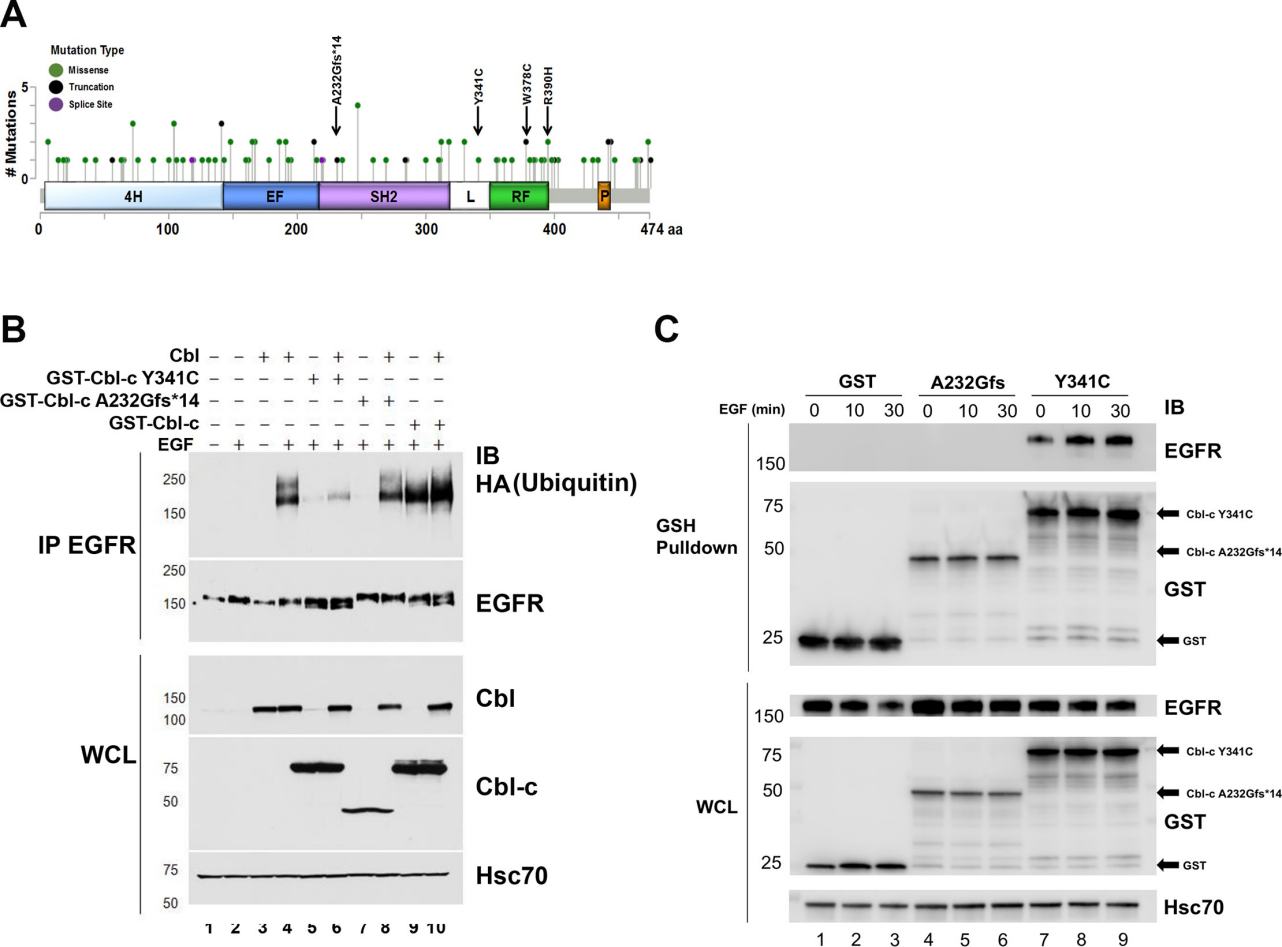
Human Cbl-c mutants Y341C and A232Gfs*14 demonstrate loss of E3 function. **(**A) Cbl-c mutations were identified in the cBioPortal for Cancer Genomics database as indicated in the cartoon. (B) Two mutants identified in lung cancer samples (a frameshift mutant in the TKB domain—A232Gfs*14, and a point mutation in the linker tyrosine—Y341C) were tested for their ability to ubiquitinate the EGFR. Cells were transfected with various combinations of WT (Cbl and GST-Cbl-c) and mutant (GST-Cbl-c Y341C and GST- Cbl-c A232Gfs*14) Cbl proteins as indicated. Cells were starved and stimulated with or without EGF for 10 min prior to isolation of protein, immunoprecipitation of EGFR, and gel electrophoresis. IP: EGFR and WCL were immunoblotted for proteins of interest. (C) GST-Cbl-c Y341C, GST-Cbl-c A232Gfs*14, or GST were cotransfected with the EGFR, the cells were starved and stimulated with EGF for 0, 10 or 30 min prior to protein isolation, and the Cbl-c proteins pulled down using GSH-agarose beads. The GST pull down and WCL lysates were probed as indicated. Molecular weight standards in kDa are indicated on the left of each panel in B and C.

We next searched the cBioPortal for Cancer Genomics database to identify Cbl-c mutations in human solid tumors [25, 26, 31]. We found 112 mutations of Cbl-c including 97 missense mutations, 13 truncating mutations, and 2 mutations affecting splice regions (Table 1 and Fig 5A). Most of these mutations occurred in epithelial malignancies–although the distribution of mutations is affected by the different numbers of tumors sequenced for each tumor type and hematological malignancies are underrepresented in this database (Table 1) [25, 26, 31]. The mutations were distributed across the Cbl-c protein (Fig 5A). Seven of the truncating mutations (black circles in Fig 5A) would encode proteins lacking all or part of the RF domain and abrogate E3 activity. In addition, there are 11 missense mutations in the linker and RF region. Several of these are similar to mutations in the equivalent amino acids in Cbl that have been shown to abrogate E3 activity (*e*.*g*., Y341C, W378C, and R390H indicated by the arrows) [32–34].

**Table 1:** Cbl-c mutations by tumor type in TCGA. Cbl-c mutations were identified in the cBioPortal for Cancer Genomics database as indicated in the table.

Cancer Type	N	Missense	Truncations	In-frame Indels	Other*
**All Sites**	**27,437**	**97**	**13**	**0**	**2**
**Hematological**	**1,393**	**7**	**0**	**0**	**0**
** Lymphoid**	**1164**	**7**	**0**	**0**	**0**
** Myeloid**	**229**	**0**	**0**	**0**	**0**
**Solid Tumors**	**26,044**	**90**	**13**	**0**	**2**
**Aerodigestive**					
** Head and Neck**	**827**	**1**	**0**	**0**	**0**
** Nasopharyngeal**	**56**	**0**	**0**	**0**	**0**
** Salivary Gland**	**214**	**0**	**0**	**0**	**0**
** Lung (Non-Small Cell)**	**2591**	**6**	**2**	**0**	**0**
** Lung (Small Cell)**	**190**	**1**	**0**	**0**	**0**
** Mesothelioma**	**109**	**0**	**0**	**0**	**0**
** Esophagus**	**557**	**4**	**0**	**0**	**0**
** Stomach**	**1267**	**7**	**1**	**0**	**0**
** Liver**	**983**	**4**	**0**	**0**	**0**
** Ampullary Carcinoma**	**160**	**2**	**0**	**0**	**0**
** Cholangiocarcinoma**	**91**	**0**	**0**	**0**	**0**
** Gall Bladder**	**32**	**0**	**0**	**0**	**0**
** Pancreas**	**832**	**0**	**0**	**0**	**0**
** Colon**	**1,462**	**11**	**3**	**0**	**0**
**Brain**	**3,200**	**9**	**0**	**0**	**0**
**Breast Cancer**	**4,110**	**5**	**2**	**0**	**0**
**Endocrine/Neuroendocrine**					
** Adrenal Cortical**	**92**	**0**	**0**	**0**	**0**
** Thyroid**	**516**	**0**	**0**	**0**	**0**
** Pancreatic Neuroendocrine**	**118**	**0**	**0**	**0**	**0**
** Pheochromocytoma**	**184**	**0**	**0**	**0**	**0**
**Genitourinary**					
** Bladder**	**705**	**1**	**0**	**0**	**0**
** Kidney**	**1,387**	**2**	**0**	**0**	**0**
** Prostate**	**2,470**	**3**	**0**	**0**	**0**
** Testicular**	**156**	**1**	**0**	**0**	**0**
**Gynecological**					
** Ovary**	**615**	**2**	**1**	**0**	**0**
** Endometrial**	**627**	**4**	**2**	**0**	**0**
** Cervical**	**309**	**2**	**1**	**0**	**0**
**Sarcoma**	**732**	**1**	**0**	**0**	**0**
**Skin**					
** Melanoma**	**900**	**23**	**1**	**0**	**2**
** Squamous**	**29**	**1**	**0**	**0**	**0**

*Mutations in splice regions that could cause missplicing

We chose one truncation mutation (A232Gfs*14) and one point mutation (Y341C) for further evaluation. The A232Gfs*14 mutation results from the insertion of a single nucleotide in the TKB domain of Cbl-c resulting in a frame shift. This mutation adds 14 amino acids encoded by the wrong reading frame and results in a truncated protein that does not contain an intact TKB or RF. The Y341C point mutation occurs in the linker tyrosine that is required for phosphorylation based activation of Cbl-c E3 activity [35]. The analogous tyrosine in Cbl is a mutation hot spot in human myeloid neoplasms [12]. Both mutants are found in lung cancers.

We tested the E3 activity as above in Figs 3 and 4 and found that both the Y341C and A232Gfs*14 mutants failed to ubiquitinate the stimulated EGFR (Fig 5B; compare lanes 4 and 9 with WT Cbl or Cbl-c respectively to lanes 5 and 7 in which the two mutant Cbl-c proteins were expressed with EGFR). The Y341C Cbl-c protein prevented ubiquitination of the activated EGFR by wild type Cbl protein (Fig 5B, compare lane 4 –Cbl alone- to lane 6 where Cbl is expressed with the Y341C mutant of Cbl-c). The truncated Cbl-c A232Gfs*14, while unable to ubiquitinate the stimulated EGFR, did not prevent ubiquitination of the EGFR by Cbl (Fig 5B, compare lane 4 to lane 8). The WT Cbl-c protein could ubiquitinate the EGFR and when cotransfected with Cbl resulted in slightly increased EGFR ubiquitination (Fig 5B, lanes 9–10).

Both Cbl-c Y341C and Cbl-c A232Gfs*14 had impaired ability to ubiquitinate the activated EGFR but only Cbl-c Y341C blocked ubiquitination of the activated EGFR by WT Cbl. Based on the mutations, Cbl-c Y341C would be expected to retain the ability to interact with the activated EGFR but Cbl-c A232Gfs*14 truncates the protein within the TKB domain and would not be expected to interact with the activated EGFR. To investigate this further, we tested their ability to interact with stimulated EGFR. HEK 293T cells transiently transfected with either GST-Cbl-c Y341C, GST-Cbl-c A232Gfs*14, or GST plasmid as a control. Cells were serum starved and then stimulated with EGF (100 ng/mL) for 0, 10 and 30 minutes. GST and GST-tagged Cbl-c mutants were pulled down using a GSH-agarose beads and probed for co-precipitated EGFR by immunoblot. The EGFR co-precipitated with GST-Cbl-c Y341C but not with either GST-Cbl-c A232Gfs*14 or GST. (Fig 5C, panel 1, compare lanes 7–9 to lanes 6–8 and 1–3, respectively) There was constitutive association of GST-Cbl-c Y341C with the EGFR that increased ~2 fold upon EGF activation (Fig 5C, panel 1, lanes 7–9). This data demonstrates that Cbl-c Y341C can interact with active EGFR, whereas Cbl-c A232Gfs*14 can not.

**Fig 6:**
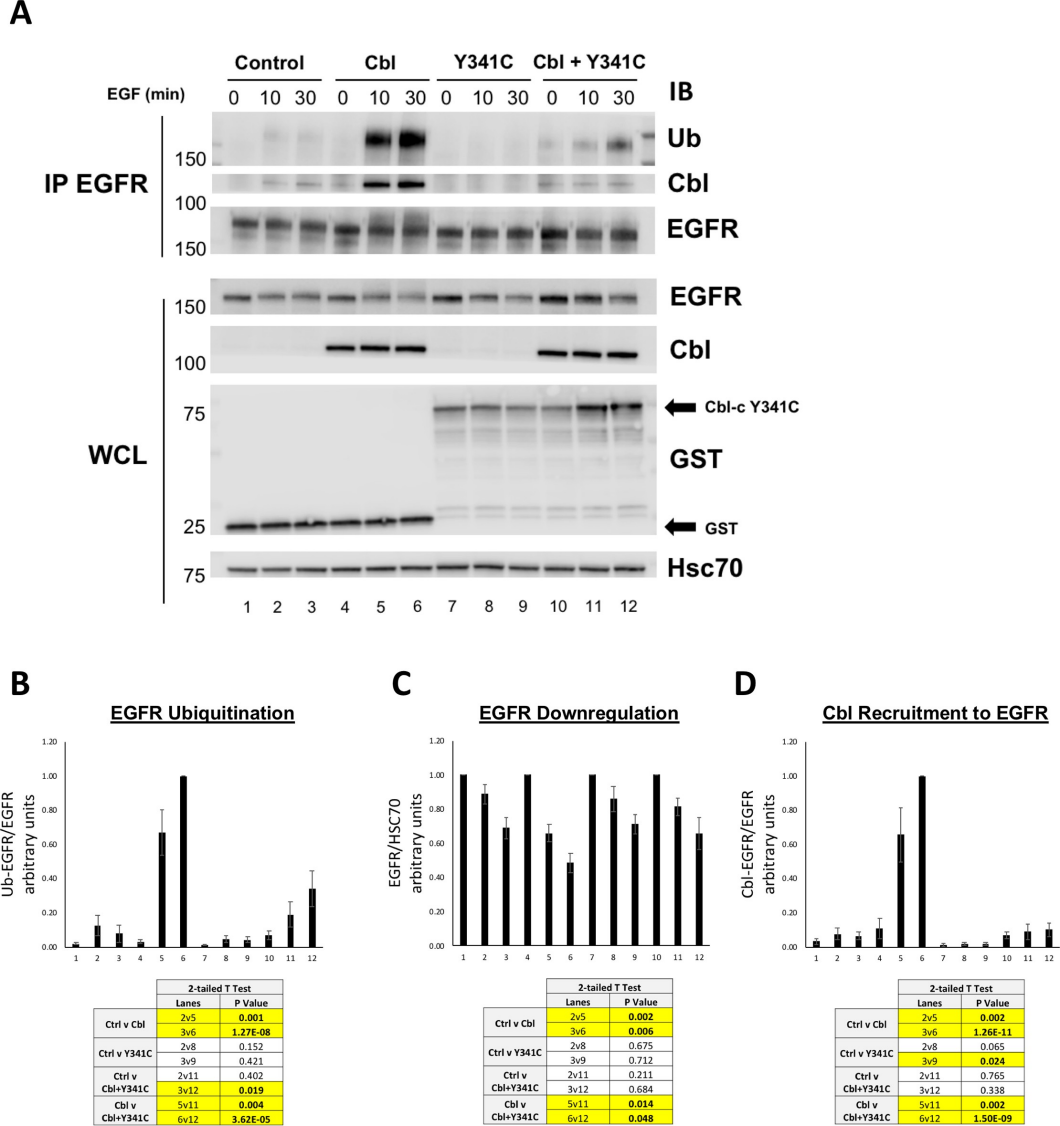
Cbl-c Y341C prevents ubiquitination and downregulation of EGFR by WT Cbl and blocks recruitment of Cbl to EGFR. (**A**) HEK 293T cells were transfected with GST (Control), WT Cbl, GST- Cbl-c Y341C, or a combination of WT Cbl and GST- Cbl-c Y341C along with EGFR. Cells were stimulated with EGF and lysates were harvested as previously described. Immunoprecipitated EGFR (IP: EGFR) and whole cell lysate (WCL) were probed for proteins as indicated. (**B**) Ubiquitination of the activated EGFR was quantified and normalized to total EGFR (**C**) EGFR levels were quantified from the WCL and normalized to Hsc70. (**D**) EGFR-associated Cbl was quantified and normalized to total EGFR (IP: EGFR). Graphs represent the average of 6 experiments +/- SE. p-values for relevant comparisons are shown in the tables accompanying the graphs. Significant comparisons (p≤0.05) are highlighted in yellow.

To further explore the dominant negative function of Cbl-c Y341C, we investigated whether or not it was capable of preventing Cbl recruitment and degradation of stimulated EGFR. HEK 293T cells were transfected with GST control, WT Cbl, GST-Cbl-c Y341C, or a combination of WT Cbl and GST-Cbl-c Y341C. Cells were serum starved and then stimulated with EGF for 0, 10 and 30 min. Immunoprecipitated EGFR and whole cell lysate samples were analyzed by immunoblotting as indicated (Fig 6A). Ubiquitination and downregulation of the activated EGFR as well as recruitment of WT Cbl to EGFR were quantified (Fig 6B, 6C and 6D, respectively).

Overexpressed WT Cbl significantly increased EGFR ubiquitination, EGFR downregulation, and was recruited to the EGFR upon EGF activation (Fig 6A–6D, compare lanes 4–6 to lanes 1–3). There were no significant differences in EGFR ubiquitination and downregulation of EGFR, when Cbl-c Y341C was transfected compared to the vector control lanes (Fig 6A, 6B and 6C, compare lanes 7–9 to lanes 1–3). There was less endogenous Cbl recruited to the activated EGFR when Cbl-c Y341C was transfected (Fig 6A and 6D, compare lanes 7–9 to lanes 1–3). Cbl-c Y341C significantly decreased EGFR ubiquitination and downregulation of activated EGFR by over-expressed WT Cbl (Fig 6A–6C, compare lanes 10–12 to lanes 4–6). Cbl-c Y341C also significantly decreased recruitment of cotransfected WT Cbl to the activated EGFR (Fig 6A and 6D, compare lanes 10–12 to 4–6).

These results indicate that Cbl-c Y341C acts as a dominant negative protein, blocking recruitment of WT Cbl to the stimulated EGFR, preventing ubiquitination of the EGFR, and inhibiting downregulation of stimulated EGFR.

**Fig 7:**
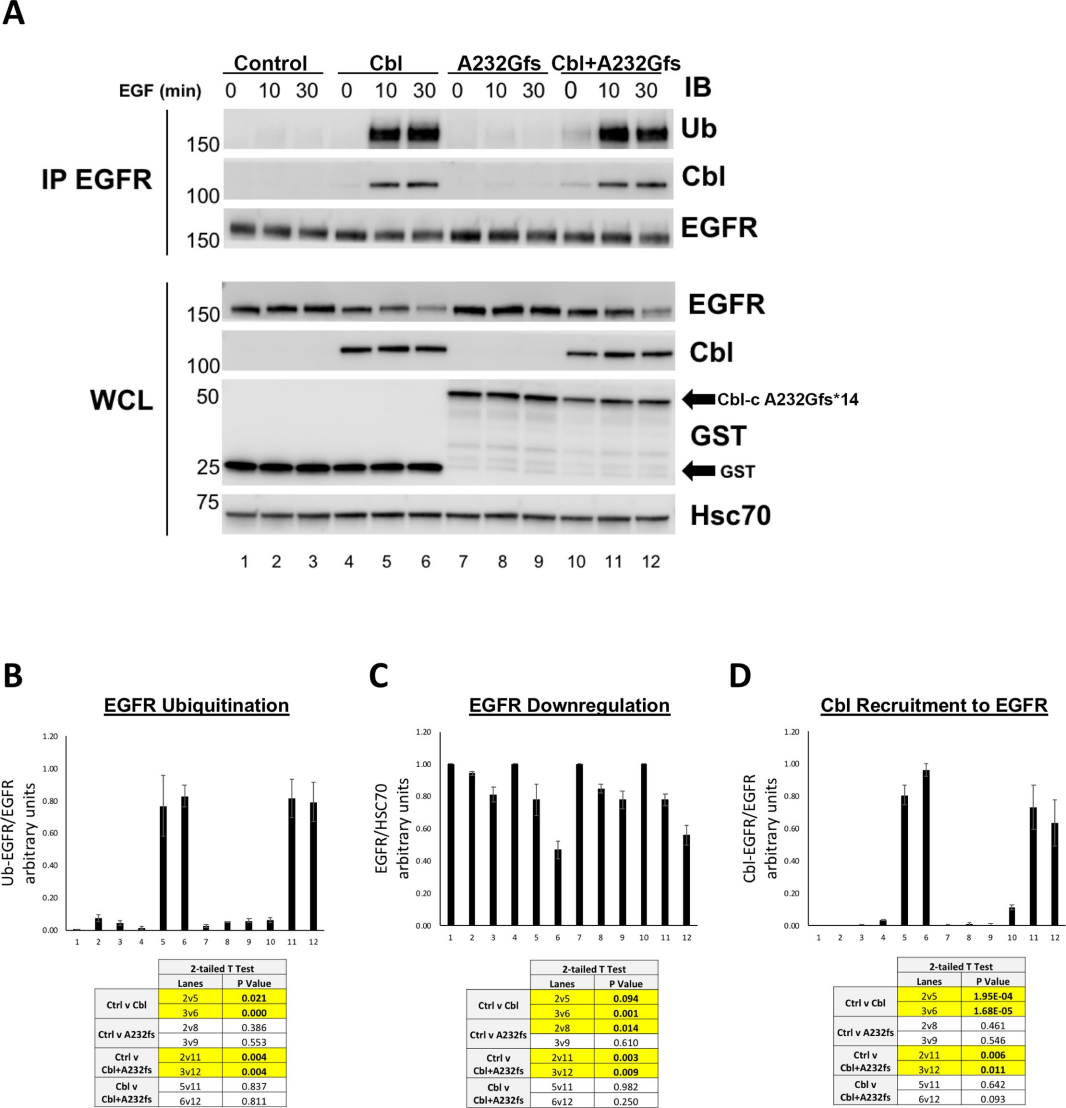
Cbl-c A232Gfs*14 does not affect ubiquitination and downregulation by WT Cbl proteins, or recruitment of WT Cbl to the stimulated EGFR. (**A**) HEK 293T cells were transfected with GST (Control), WT Cbl, GST-Cbl-c A232Gfs*14 (A232Gfs), or a combination of WT Cbl and GST- Cbl-c A232Gfs*14. All cells were cotransfected with EGFR. Cells were stimulated with EGF and lysates were harvested as described above. Immunoprecipitated EGFR (IP: EGFR) and whole cell lysate (WCL) were probed for proteins as indicated. (**B**) Ubiquitination of the activated EGFR was quantified and normalized to total EGFR (**C**) EGFR levels were quantified from the WCL and normalized to Hsc70. (**D**) EGFR-associated WT Cbl was quantified and normalized to total EGFR (IP: EGFR). Graphs represent mean +/- SE for 3 experiments. p-values for relevant comparisons are shown in the tables accompanying the graphs. Significant comparisons (p≤0.05) are highlighted in yellow.

GST-Cbl-c A232Gfs*14 showed no E3 activity for the activated EGFR (Fig 5B, lane 7) and did not associate with the activated EGFR (Fig 5C, lanes 4–6). To further evaluate the effects of the truncated Cbl-c mutant on the ability of WT Cbl to ubiquitinate, downregulate and be recruited to the activated EGFR, we transfected either GST control, WT Cbl, GST-A232Gfs*14 Cbl-c, or a combination of Cbl and GST-A232Gfs*14 Cbl-c into HEK 293T cells (Fig 7A). Cells were serum starved and stimulated with EGF as above. Ubiquitination of EGFR, EGFR downregulation, and Cbl recruitment to EGFR was evaluated as above (Fig 7A–7D).

As seen above, expression of WT Cbl induced significantly increased ubiquitination of the activated EGFR compared to the empty vector control (Fig 7A and 7B, compare lanes 4–6 to lanes1-3), whereas GST-Cbl-c A232Gfs*14 did not increase ubiquitination of the activated EGFR (Fig 7A and 7B, compare lanes 7–9 to lanes1-3). There was no significant difference in ubiquitination of the activated EGFR in cells cotransfected with WT Cbl and GST-Cbl-c A232Gfs*14 as compared to over-expressed WT Cbl alone (Fig 7A and 7B, compare lanes 10-12to lanes 4–6). EGFR downregulation was increased by expression of WT Cbl and this was not inhibited by cotransfection of Cbl-c A232Gfs*14 (Fig 7A and 7C, compare lanes 4–6 to lanes 10–12).WT Cbl recruitment to EGFR was the same when WT Cbl was expressed alone or co-expressed with GST-Cbl-c A232Gfs*14 (Fig 7A and 7D, compare lanes 10–12 to lanes 4–6).

Overall, the data indicates that Cbl-c A232Gfs*14 does not interfere with WT Cbl recruitment, ubiquitination, and subsequent downregulation of stimulated EGFR.

## Discussion

Cbl-c is a member of the Cbl Ring Finger E3 family [14, 17]. Like other members of the Cbl family, Cbl-c can negatively regulate RTK signaling by ubiquitinating and downregulating activated RTKs such as the EGFR and RET [18, 21, 24, 36, 37]. Cbl-c is expressed predominantly in epithelial cells while Cbl and Cbl-b are expressed more ubiquitously [13, 14, 38]. Mutations in Cbl that abrogate E3 activity and create a dominant negative Cbl protein that can block interaction between other Cbl proteins and the target RTKs have been described in ~5% of myeloid neoplasms [12]. In this work, we identified mutations in E3 Cbl-c in a murine breast cancer and in human solid tumors that would inactivate the E3 activity of Cbl-c and in some cases, create dominant negative proteins that could interfere with the activity of endogenous wild type Cbl proteins.

The murine mutation (Cbl-c Δex7) occurred in a mammary tumor that arose in a genetically engineered mouse model–the C3(1) SV40 Large T antigen transgenic mouse model of breast cancer [28, 29]. The cDNA of Cbl-c that was cloned from this tumor expresses a form of Cbl-c that is lacking exon 7, resulting in an in-frame deletion of the distal part of the linker region and the proximal part of the RF domain (Fig 1). Similar in-frame deletions of part or all of the RF in Cbl are seen in murine lymphomas and human myeloid neoplasms (reviewed in [12]). Several mechanisms have been described for the generation of these in-frame deletions in the mRNA including point mutations, insertions, or deletions in the splice donor and acceptor sites surrounding linker and RF exons and larger deletions encompassing one or more exons [12]. The mutant Cbl-c cDNA was sequenced as part of a large-scale cDNA sequencing project and submitted to GenBank in 2005 [39]. Since the tumor, mRNA, cDNA library, or the gDNA derived from the tumor are no longer available, we cannot definitively identify the mechanism by which this in-frame deletion of exon 7 occurred.

The mutation in Cbl-c likely arose as a second hit in a breast tumor that was driven by Tag [28, 29]. Consistent with this, there is no evidence of deletion by Southern analysis (Fig 1C) or small INDELs or mutations by sequence analysis in the gDNA of the parental or transgenic mouse strains. Further, the Cbl-c Δex7 mutant did not transform NIH 3T3 cells in a foci assay by itself but increased the frequency of transformed cells when cotransfected with Tag (Fig 2). We did not identify any other mutations in Cbl-c in 21 additional tumors, indicating that such Cbl-c mutations are rare in this setting. Rare second hit mutations in Cbl have been seen in an animal model of myelodysplastic syndrome [40].

The deletion in the Cbl-c Δex7 cDNA encodes a protein that lacks the critical linker tyrosine that is phosphorylated to activate the E3 activity of the Cbl proteins [35, 41, 42] and the first 4 zinc coordinating amino acids of the RF (Fig 1). As expected, the Cbl-c Δex7 protein lacks E3 activity as demonstrated by the lack of increase in ubiquitination of the activated EGFR when cotransfected with Cbl-c Δex7 compared to the wild type Cbl-c protein (Fig 3A). Cbl proteins interact with the activated EGFR via their N-terminal TKB domain [1] which is intact in the Cbl-c Δex7 protein. The mutant can interact with the EGFR via the TKB domain but is unable to ubiquitinate the EGFR. The Cbl-c Δex7 mutant prevents ubiquitination of the activated EGFR by WT Cbl-c (Fig 3B). Thus, the Cbl-c Δex7 acts as a dominant negative protein and inhibits ubiquitination of the EGFR by the wild type Cbl-c (Fig 3B).

The Cbl proteins have been shown to interact with and negatively regulate many RTKs and non-RTK signaling pathways [1, 17] so that the dominant negative function of Cbl-c Δex7 could have broad effects by inhibiting the function of other Cbl proteins. To test if Cbl-c Δex7 could similarly inhibit other Cbl proteins, we over-expressed WT Cbl in the presence and absence of Cbl-c Δex7 and measured ubiquitination and downregulation of EGF-stimulated EGFR and Cbl association with activated EGFR. Our data show that Cbl-c Δex7 was able to inhibit WT Cbl recruitment to EGFR and as a result inhibit ubiquitination of EGFR (Fig 4B–4D). We also demonstrate that Cbl-c Δex7 prevents downregulation of activated EGFR, which could stimulate proliferation in EGFR-dependent epithelial cancer cells. Our findings are also consistent with other dominant negative Cbl mutations and their inhibitory effects on endogenous protein function.

We explored the spectrum of mutations of Cbl-c in human tumors in the cBioPortal for Cancer Genomics database (Table 1, Fig 5) [25, 26, 31]. The majority of the mutations occur in solid tumors but the number of hematological malignancies in this data base is relatively low (Table 1). Overall, mutations in Cbl-c are infrequent (112 or <0.5%) and were most frequent in melanoma, colorectal cancer, and endometrial cancer (2.9, 0.96, and 0.95%, respectively) (Table 1). All of these tumor types have relatively high mutational loads with melanoma the highest of all tumors so that this high frequency may reflect a high overall mutation rate and not indicate a specific role for Cbl-c [43]. For comparison, mutations of Cbl and Cbl-b in the same database were more frequent (199 and 210 for Cbl and Cbl-b, respectively) (S1 Fig, S1 Table, and S2 Table). This may reflect the larger size of both the exons and genomic loci for Cbl and Cbl-b compared to Cbl-c. The coding regions of Cbl and Cbl-b are ~2 fold larger than that of Cbl-c (3081, 3971, vs. 1574 bp, respectively) and the genomic loci for Cbl and Cbl-b are considerably larger than that of Cbl-c (~110 kb, ~220 kb, vs. ~20 kb, respectively) [30]. Similar to Cbl-c, the tumors with the most frequent mutations in Cbl and Cbl-b were again melanoma, endometrial cancer, colorectal cancer, and non-small cell lung cancer (S1 Table and S2 Table). Mutations of Cbl have been previously described in non-small cell lung cancer [44]. Distinct from Cbl-c (or Cbl-b), mutations of Cbl have been found in ~5% of myeloid neoplasms including myelodysplastic syndrome, myelofibrosis, refractory anemia with excess blasts, de novo and secondary acute myeloid leukemia, atypical chronic myelogenous leukemia, CML in blast crisis, chronic myelomonocytic leukemia, and juvenile myelomonocytic leukemia (reviewed in [1, 12]). The cBioPortal for Cancer Genomics database has few of these neoplasms so that this was not seen in our evaluation. However, evaluation of >2000 myeloid neoplasms has found Cbl mutations in approximately 5% of these neoplasms [1, 12]. Mutations of Cbl-c and Cbl-b in myeloid neoplasms are much less common (~5 mutations in each reported) and their significance in myeloid neoplasms is unclear [1, 12].

Many of the missense mutations of Cbl-c seen in solid tumors are likely to be passenger mutations. However, 11 of the 97 missense mutations occur in the linker region or the RF and could potentially disrupt E3 function (Fig 5). Several of these mutations occur in amino acids known to be important for E3 function including one mutation at the conserved linker tyrosine (Y341C), two point mutations at a tryptophan in the RF (W378C), and a mutation in the arginine that is immediately after the last zinc coordinating cysteine (R390H). Mutations of each of the corresponding amino acid residues in Cbl abrogate E3 activity [32, 34, 35, 41, 42]. There are 7 truncation mutations that would disrupt the Cbl-c protein before or within the RF and would thereby be predicted to abrogate E3 function (Fig 5). Finally, there are two splice site mutations that could potentially lead to deletions or frame shifts that could disrupt the E3 activity (Fig 5). Altogether, 20 of the 112 mutations may lead to functional disruption of the protein. Analysis of the Cbl and Cbl-b mutations found in the cBioPortal for Cancer Genomics database found that there was a similar fraction of the overall mutations that might disrupt E3 function (33 and 15 for Cbl and Cbl-b, respectively).

We tested the ability of one Cbl-c truncation mutation (A232Gfs*14) and one Cbl-c point mutation (Y341C) to ubiquitinate the activated EGFR in cells and as predicted both mutations abrogate E3 activity (Fig 5). However, the mutations behaved differently in their ability to act in a dominant negative manner. Only Cbl-c Y341C acted as a dominant negative protein, whereas Cbl-c A232Gfs*14 did not disrupt ubiquitination by WT Cbl proteins (Figs 5B, 6 and 7). The Cbl-c Y341C mutation leaves the TKB domain intact, and so this protein can interact with the activated EGFR (Fig 5C). While Cbl-c Y341C fails to ubiquitinate the EGFR, it also prevents ubiquitination by blocking recruitment of the WT Cbl protein to its target substrate (Fig 6D). This inhibition of E3 function by the point mutation subsequently blocks downregulation of stimulated EGFR (Fig 6C). In contrast, the truncation mutation disrupts the Cbl-c protein within the TKB and thus the Cbl-c A232Gfs*14 mutant cannot interact with the EGFR (Fig 5C). As a result, expression of Cbl-c A232Gfs*14 does not disrupt recruitment of WT Cbl to the activated EGFR, does not block ubiquitination by WT Cbl, and does not inhibit downregulation of the activated EGFR by WT Cbl. (Fig 7). These data suggest that mutations or truncations that disrupt the RF but leave the TKB domain intact are more likely to have significant effects due to their ability to interfere with the wild type Cbl proteins in the cell.

Overall these data identify mutations of Cbl-c that occur in human solid tumors and disrupt E3 activity. To our knowledge this is the first description of Cbl-c mutations that may contribute to the development of human solid tumors. Moreover, similar mutations are found in Cbl and Cbl-b from solid tumors suggesting that Cbl-c and the Cbl proteins in general may contribute to the pathogenesis of human solid tumors.

## Supporting information

S1 Fig Cbl and Cbl-b mutations in human tumors. (TIF)

S1 Table Cbl mutations by tumor type in TCGA. (PDF)

S2 Table Cbl-b mutations by tumor type in TCGA. (PDF)
