# Supplementary figures and images for: Loss of function Cbl-c mutations in solid tumors
Source: PLoS One. 2019 Jul 1;14(7):e0219143. doi: 10.1371/journal.pone.0219143 (PMC6602201; doi:10.1371/journal.pone.0219143)

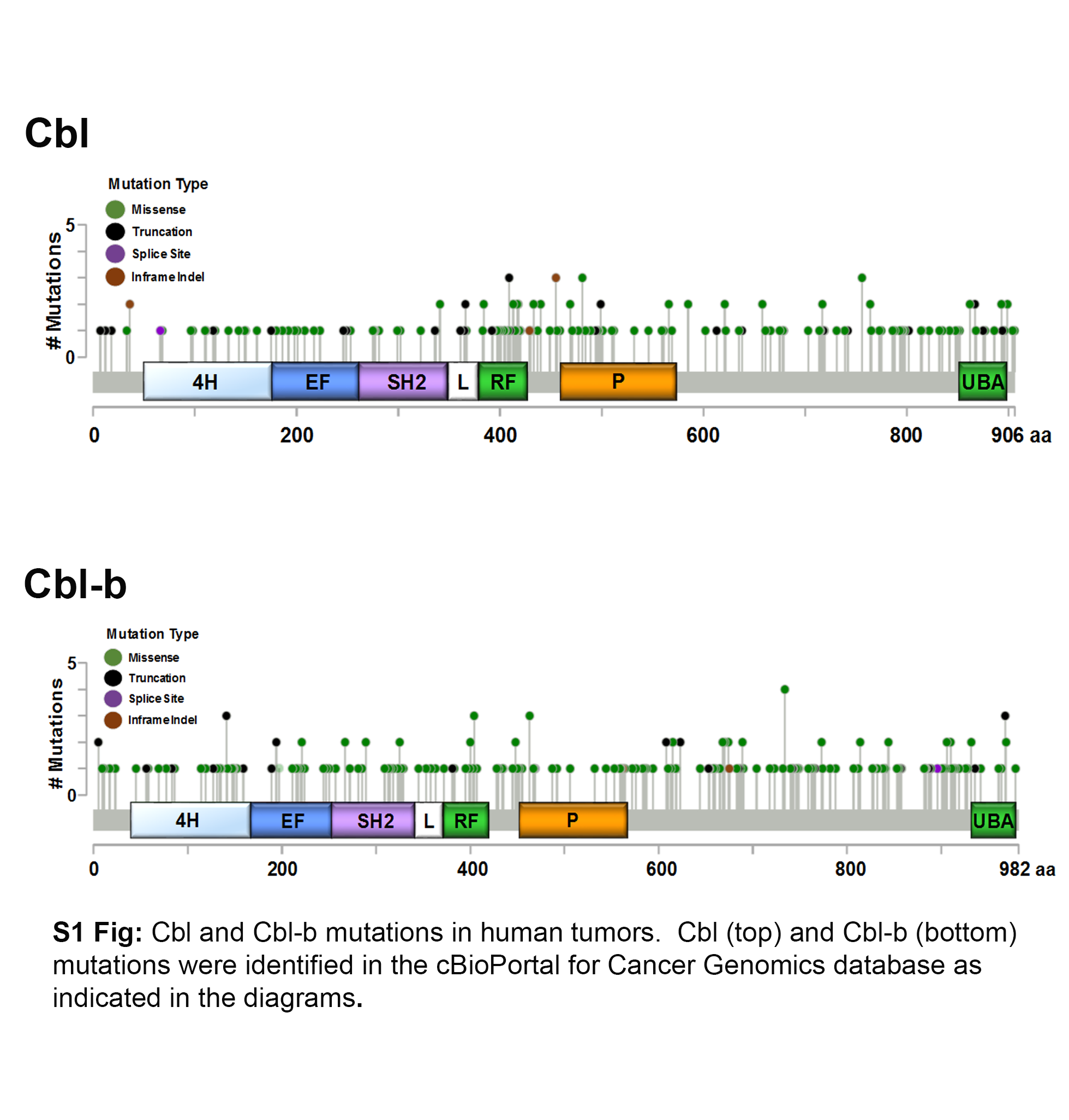

Supplement: S1 Fig — (TIF) [file pone.0219143.s001.tif]
