# Supplementary material for: Loss of function Cbl-c mutations in solid tumors
Source: PLoS One. 2019 Jul 1;14(7):e0219143. doi: 10.1371/journal.pone.0219143 (PMC6602201; doi:10.1371/journal.pone.0219143)
Supplement: S1 Table — (PDF) [file pone.0219143.s002.pdf]

S1 Table: Cbl Mutations by Tumor Type in TCGA

Cancer Type	N	Missense	Truncations	Inframe Indels	Other*
All Sites	27,437	155	37	5	2
Hematological	1,393	4	1	0	0
Lymphoid	1164	2	0	0	0
Myeloid	229	2	1	0	0
Solid Tumors	26,044	151	36	5	2
Aerodigestive					
Head and Neck	827	5	0	1	0
Nasopharyngeal	56	0	0	0	0
Salivary Gland	214	0	0	0	0
Lung (Non-Small Cell)	2591	25	10	2	0
Lung (Small Cell)	190	7	0	0	0
Mesothelioma	109	0	0	0	0
Esophagus	557	6	0	0	0
Stomach	1267	7	3	2	0
Liver	983	7	2	0	0
...Ampullary Carcinoma	160	0	0	0	0
Cholangiocarcinoma	91	0	0	0	0
Gall Bladder	32	0	0	0	0
Pancreas	832	3	1	0	0
Colon	1,462	17	2	0	0
Brain	3,200	7	5	0	1
Breast Cancer	4,110	6	1	0	1
Endocrine/Neuroendocrine					
Adrenal Cortical	92	1	0	0	0
Thyroid	516	0	0	0	0
Pancreatic Neuroendocrine	118	0	0	0	0
Pheochromocytoma	184	0	0	0	0
Genitourinary					
Bladder	705	8	1	0	0
Kidney	1,387	4	0	0	0
Prostate	2,470	8	1	0	0
Testicular	156	0	1	0	0
Gynecological					
Ovary	615	0	0	0	0
Endometrial	627	12	3	0	0
Cervical	309	1	0	0	0
Sarcoma	732	1	1	0	0
Skin					
Melanoma	900	22	5	0	0
Squamous	29	4	0	0	0

*Mutations in splice regions that could cause missplicing
