# Supplementary material for: Loss of function Cbl-c mutations in solid tumors
Source: PLoS One. 2019 Jul 1;14(7):e0219143. doi: 10.1371/journal.pone.0219143 (PMC6602201; doi:10.1371/journal.pone.0219143)
Supplement: S2 Table — (PDF) [file pone.0219143.s003.pdf]

S2 Table: Cbl-b Mutations by Tumor Type in TCGA

Cancer Type	N	Missense	Truncations	Inframe Indels	Other*
All Sites	27,437	180	26	2	2
Hematological	1,393	4	2	0	0
Lymphoid	1164	4	2	0	0
Myeloid	229	0	0	0	0
Solid Tumors	26,044	176	24	2	2
Aerodigestive					
Head and Neck	827	5	1	0	0
Nasopharyngeal	56	0	0	0	0
Salivary Gland	214	0	0	0	0
Lung (Non-Small Cell)	2591	36	6	1	0
Lung (Small Cell)	190	4	1	0	0
Mesothelioma	109	0	0	0	0
Esophagus	557	4	2	0	0
Stomach	1267	4	1	0	0
Liver	983	4	2	0	1
...Ampullary Carcinoma	160	2	1	0	0
Cholangiocarcinoma	91	0	0	0	0
Gall Bladder	32	0	0	0	0
Pancreas	832	3	0	0	0
Colon	1,462	23	3	0	0
Brain	3,200	0	0	0	0
Breast Cancer	4,110	13	0	0	0
Endocrine/Neuroendocrine					
Adrenal Cortical	92	1	0	0	0
Thyroid	516	0	0	0	0
Pancreatic Neuroendocrine	118	0	0	0	0
Pheochromocytoma	184	0	0	0	0
Genitourinary					
Bladder	705	10	1	0	0
Kidney	1,387	5	1	0	0
Prostate	2,470	5	0	0	0
Testicular	156	0	0	0	0
Gynecological					
Ovary	615	1	0	0	0
Endometrial	627	13	3	0	0
Cervical	309	4	0	0	0
Sarcoma	732	3	0	0	0
Skin					
Melanoma	900	38	2	1	1
Squamous	29	1	0	0	0

*Mutations in splice regions that could cause missplicing
